# Identification of common genetic characteristics of rheumatoid arthritis and major depressive disorder by bioinformatics analysis and machine learning

**DOI:** 10.3389/fimmu.2023.1183115

**Published:** 2023-06-21

**Authors:** Wen Jiang, Xiaochuan Wang, Dongxia Tao, Xin Zhao

**Affiliations:** ^1^ Department of Orthopedics, First Hospital of China Medical University, Shenyang, Liaoning, China; ^2^ Nurse Department, The First Hospital of China Medical University, Shenyang, Liaoning, China; ^3^ Department of Operation Room, The First Hospital of China Medical University, Shenyang, Liaoning, China

**Keywords:** rheumatoid arthritis, major depressive disorder, differentially expressed genes, machine learning, diagnosis, immune infiltration

## Abstract

**Introduction:**

Depression is the most common comorbidity of rheumatoid arthritis (RA). In particular, major depressive disorder (MDD) and rheumatoid arthritis share highly overlapping mental and physical manifestations, such as depressed mood, sleep disturbance, fatigue, pain, and worthlessness. This overlap and indistinguishability often lead to the misattribution of physical and mental symptoms of RA patients to depression, and even, the depressive symptoms of MDD patients are ignored when receiving RA treatment. This has serious consequences, since the development of objective diagnostic tools to distinguish psychiatric symptoms from similar symptoms caused by physical diseases is urgent.

**Methods:**

Bioinformatics analysis and machine learning.

**Results:**

The common genetic characteristics of rheumatoid arthritis and major depressive disorder are EAF1, SDCBP and RNF19B.

**Discussion:**

We discovered a connection between RA and MDD through immune infiltration studies: monocyte infiltration. Futhermore, we explored the correlation between the expression of the 3 marker genes and immune cell infiltration using the TIMER 2.0 database. This may help to explain the potential molecular mechanism by which RA and MDD increase the morbidity of each other.

## 1 Introduction

The Global Burden of Disease 2019 study indicates that depressive disorders are one of the ten most important drivers of the increase in the world’s disease burden (1). Globally, there were almost 20 million prevalent cases of rheumatoid arthritis (RA), which is reported in GBD 2017 (2). Depression is the most common comorbid disorder associated with RA (3). A meta-analysis of 11 cohort studies involving a total of 39,130 RA patients and 7,802,230 patients in the control group showed that patients with RA have a 47% higher risk of depression than the control group (4). Another meta-analysis of 72 reports involving a total of 13,189 RA patients shows that the prevalence of depression in patients with RA was 34.2% (95% CI 25%,44%) and 14.8% (95% CI 12%,18%), respectively, according to the Hospital Anxiety and Depression Scale (HADS) threshold of 8 and 11. The prevalence of major depressive disorder (MDD) was 16.8% (95% CI 10%,24%) according to DSM diagnostic criteria (5). In this meta-analysis, depression was defined in 40 different ways. The results vary greatly due to the use of different screening questionnaires or different thresholds of the same screening questionnaire. In addition, symptoms of RA caused by physical and mental disorders often have indistinguishable overlap (6). Fatigue, loss of appetite, pain, or sleep disorders in people with RA may be mistakenly attributed to physical illness rather than mental disorders. On the contrary, if these symptoms are generally attributed to mental disorders, it will overestimate the prevalence of depression in people with RA. The comorbidity symptoms bring great difficulties for the diagnosis of depression in patients with RA, and this difficulty cannot be alleviated by optimizing screening questionnaires. Therefore, there is an urgent need to find the common pathogenesis of RA and MDD, so as to provide a theoretical basis for the development of new treatments for the common pathogenesis.

A number of evidence shows that a variety of physiological functions of patients with depression are affected, including inflammation (7, 8), neurotransmitters (9, 10), neuroendocrine function (11, 12), neuro nutrition (13) and so on. Each physiological function can be evaluated at different biological levels, including genomics, epigenomics, transcriptome, proteomics, metabonomics and so on. Different histological studies of various tissues may contribute to a more accurate diagnosis of depression in patients with RA.

In this study, we found several key cellular signaling pathways and gene networks related to RA and MDD. In addition, we identified three key genes through machine learning, which may can explain the common pathogenesis of RA and MDD. We also studied the immune infiltration of RA and MDD respectively and found that monocyte infiltration may be an important mechanism for connecting RA and MDD. In addition, we used the TIMER 2.0 database and Cibersort algorithm to explore the potential correlation between the expression of this three genes and immune cell infiltration.

## 2 Materials and methods

### 2.1 Data download, processing and differentially expressed gene analysis

**Figure 1:**
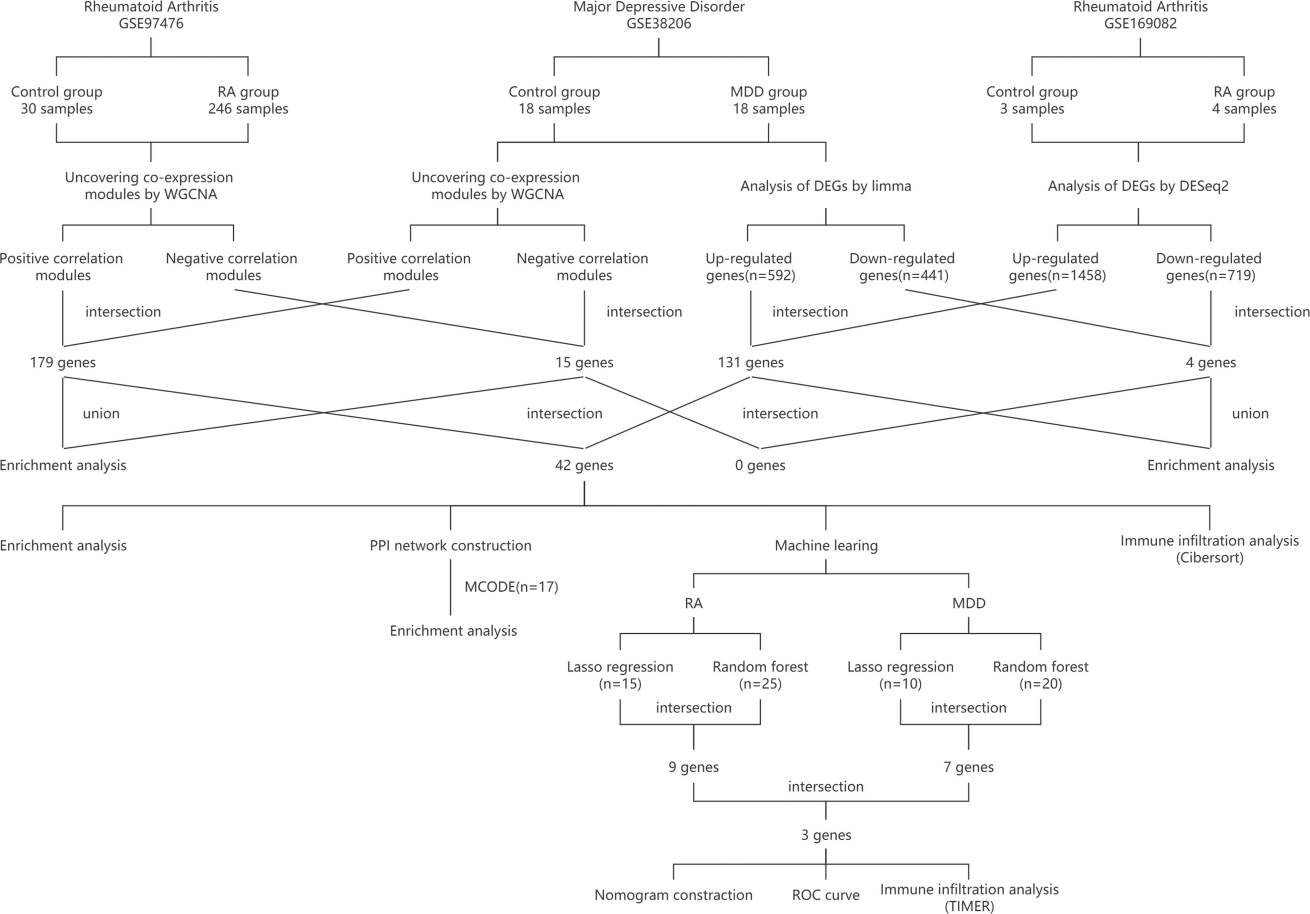
Study flowchart. RA, rheumatoid arthritis; MDD, major depressive disorder; GSE, gene expression omnibus series; WGCNA, weighted gene co-expression network analysis; limma, linear models for microarray data; DEGs, differentially expressed genes; PPI network, protein-protein interaction network; MCODE, molecular complex detection; ROC curve, receiver operating characteristic curve; TIMER, Tumor Immune Estimation Resource.

In **Figure 1**, the study flowchart is shown. Three raw datasets were downloaded from the GEO database (https://www.ncbi.nlm.nih.gov/geo/), one for MDD and two for RA patients. Details about the dataset are presented in **Table 1**, including the platform, sample groups, and numbers. GEOquery downloads expression sets and clinical data first. To determine the expression of a gene identified by multiple probes, the max value was selected. Lastly, DEGs were identified using DEseq2 and limma package after checking |log2 fold change (FC)| > 1 for GSE169082 and |log2 fold change (FC)| > 0.4 for GSE38206 and adjusted p-value of less than 0.05 (14, 15).

**Table 1:** Basic information of GEO datasets used in the study.

GSE series	Source type	Sample size		Platform
		**Control**	**Rheumatoid arthritis**	
GSE97476	PBMC	30	246	GPL10904
GSE169082	PBMC	3	4	GPL20795
		**Control**	**Major depressive disorder**	
GSE38206	PBMC	18	18	GPL13607

### 2.2 Weighted gene co-expression network analysis and module gene selection

To explore the correlation among genes, the WGCNA strategy was adopted (16). First, the top 5000 genes in the standard deviation were used for WGCNA. A scale-free co-expression network was constructed by removing unqualified genes and samples from the expression matrix using the goodSamplesGenes function. Thirdly, adjacency was determined using co-expression similarity-derived “soft” thresholding powers (β). To determine gene ratio and dissimilarity, the adjacency was converted into a topological overlap matrix (TOM). Using hierarchical clustering and a dynamic tree cut function, modules were detected in the fourth step. Using average linkage hierarchical clustering, genes with identical expression profiles were classified into gene modules, using a TOM-based dissimilarity metric and a minimum gene group size. For the fifth step, the dissimilarity of module eigengenes was calculated, a cut line for the module dendrogram was chosen, and several modules were combined. After visualizing the eigengene network, WGCNA analysis was used to identify important modules in RA and MDD. Significant module genes were selected to be analyzed.

### 2.3 Analysis of functional enrichment

Gene Ontology (GO) provides structured, computable information concerning gene functions (17). A widely used database for studying gene functions is the Kyoto Encyclopedia of Genes and Genomes (KEGG) (18). The ClusterProfiler R package was used to analyze functional enrichment (19), and The results of enrichment analysis were visualized with ggplot2. Adjusted p-Value <0.05 was set as the criteria. Based on the intersection of DEGs and significant module genes, GO and KEGG analyses were performed four times, the intersection of common DEGs and common module genes and the genes filtered by MCODE in cytoscape.

### 2.4 Protein–protein interaction network construction

The String database(version 11.5; www.string-db.org) was used to establish a network of protein–protein interactions (PPIs) between protein-coding genes (20), with the minimum required interaction score is 0.400. We used Cytoscape software to modify String images, and the MCODE to identify interacted genes (21). In a subsequent analysis, all genes that could interact with each other in the PPI network were selected.

### 2.5 Machine learning

RA and MDD candidate genes were further filtered using two machine learning algorithms. LASSO regression method improves predictive accuracy by selecting a variable. Additionally, it is a regression technique for selecting variables and regularizing them to improve a statistical model’s predictive accuracy and comprehensibility. In addition to the benefits of not being limited by variables, RF provides better accuracy, sensitivity, and specificity as well, which can be used to predict continuous variables and make accurate forecasts. “glmnet” (22) and “randomForest” (23) R packages were used to perform LASSO regression and RF analysis. LASSO and RF intersection genes were considered candidate hub genes for RA or MDD.

### 2.6 Receiver operating characteristic evaluation and nomogram construction

The construction of nomograms is valuable for RA or MDD diagnosis in clinical settings. In order to construct the nomogram, the “rms” R package was used. “Points” indicates the scores of candidate genes, and “Total Points” indicates the sum of all the scores. In addition, the ROC was established to evaluate the diagnostic value of RA and MDD nomograms separately, and the pROC package calculated the area under the curve (AUC) and 95% confidence interval (CI) to quantify its value (24). The ideal diagnostic value was considered to be AUC > 0.7. Assess the importance of genes based on this.

### 2.7 Immune infiltration analysis

Cibersort, a computational method to identify the proportion of different immune cells using tissue gene expression profiles, was used to determine the proportion of immune cells in RA and MDD (25). The TIMER database (https://cistrome.shinyapps.io/timer/) includes 10,897 samples across 32 cancer types from The Cancer Genome Atlas (TCGA) to estimate the abundance of immune infiltrates (26). The “Cibersort” R script and TIMER 2.0 database were used to perform immune cell infiltration analysis. Using the heatmap R package, a heatmap was created showing 22 types of immune cells infiltrating the body. In the boxplot, we visualized the differences between patient and control groups regarding the proportion of different types of immune cells. The relations between hub gene expression and immune cells were visualized via the scatterplot and linear fitting. To compare the proportions of immune cells between controls and patients, Wilconxon tests were applied, and p-value <0.05 was considered statistically significant (27).

### 2.8 Statistical analysis

The statistical analysis was all calculated by R 4.2.1. Based on pROC, the ROC curve was constructed and the AUC (95%CI) calculated. Wilcoxon test was applied to compare proportions of different immune cells between control and patient groups in RA and MDD separately via Base package. Linear fitting and scatterplot was done by ggstatsplot package (28). It was considered statistically significant when the P-Value was below 0.05.

## 3 Results

### 3.1 Analysis of weighted gene coexpression networks and identification of key modules

**Figure 2:**
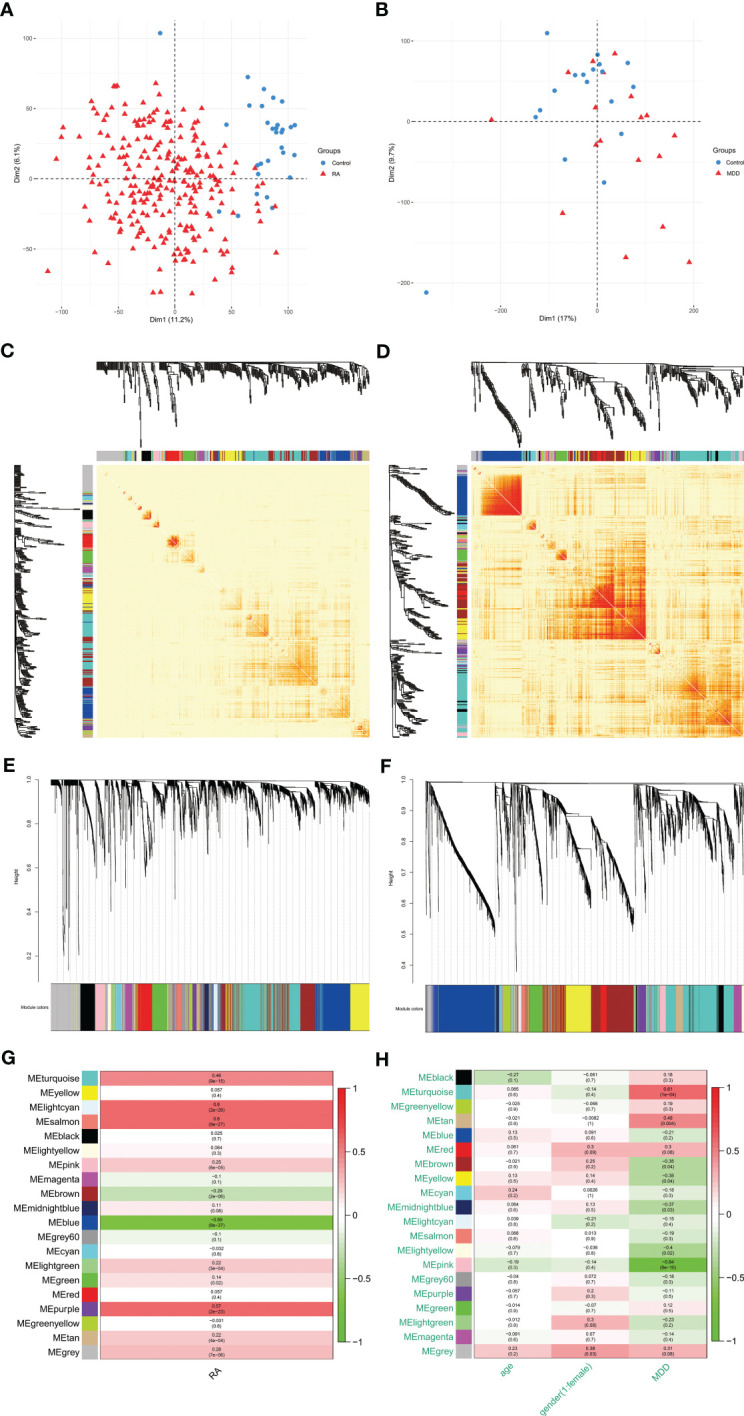
PCA and WGCNA of RA dataset (GSE97476) and MDD dataset (GSE38206). **(A)** PCA of genes of RA dataset. **(B)** PCA of genes of MDD dataset. **(C)** Modular gene clustering heat map of genes of RA dataset. Each branch of the tree represents a gene, and the darker the color of each point (white, yellow, red) represents the stronger the correlation between the two genes corresponding to the row and column. **(D)** Modular gene clustering heat map of genes of MDD dataset. **(E)** Clustering dendrogram of genes of RA dataset. The module merging threshold is set to 0.25 and the minimum number of module genes is 50. **(F)** Clustering dendrogram of genes of MDD dataset. The module merging threshold is set to 0.25 and the minimum number of module genes is 50. **(G)** Module–trait associations of genes of RA dataset. Each row corresponds to a module, and each column corresponds to a trait. Each cell contains the corresponding correlation and P value. **(H)** Module–trait associations of genes of MDD dataset. PCA, principal component analysis; WGCNA, weighted gene co-expression network analysis; RA, rheumatoid arthritis; MDD, major depressive disorder; GSE, gene expression omnibus series.

Using PCA to analyze the gene expression patterns of different groups of GSE97475 and GSE38206, there are certain differences between the disease group and the control group **Figures 2A, B**. The most correlated module was identified using WGCNA in GSE97476 and GSE38206. For GSE97476, we chose β=7 (scale-free R2 = 0.9) as a “soft” threshold based on scale independence and average connectivity (**Figure S1A**). **Figures 2C, E** depicts the clustering dendrogram of the RA and associated control groups. Based on this power, 19 gene coexpression modules (GCMs) were generated, and are shown in **Figure 2G**. Four modules “lightcyan”, “salmon”, “blue” and “purple” have high association with RA and were selected as RA-related modules(lightcyan module: r=0.60,p=2e-26; salmon module: r=0.60,p=9e-27;blue module: r=-0.69,p=8e-37,purple module: r = 0.57, p=2e-23).The lightcyan, salmon and purple modules were positively correlated with RA, included 71, 88 and 109 genes, respectively. The blue modules were negatively correlated with RA, included 636 genes. For GSE38206, we chose β=6(scale-free R2 = 0.9) as a “soft” threshold based on scale independence and average connectivity (**Figure S1B**). The clustering dendrogram of MDD and controls is shown in **Figures 2D, F**. Similarly, a total of 19 modules were identified in GSE38206 (**Figure 2H**). Two modules “turquoise” and “pink” have high association with MDD and were selected as MDD-related modules (turquoise module: r=0.61, p=1e-04; pink module: r=-0.84, p=8e-10). The turquoise module are positively correlated with MDD, included 1064 genes. The pink modules are negatively correlated with MDD, included 131 genes.

### 3.2 Genes expressed differentially

**Figure 3:**
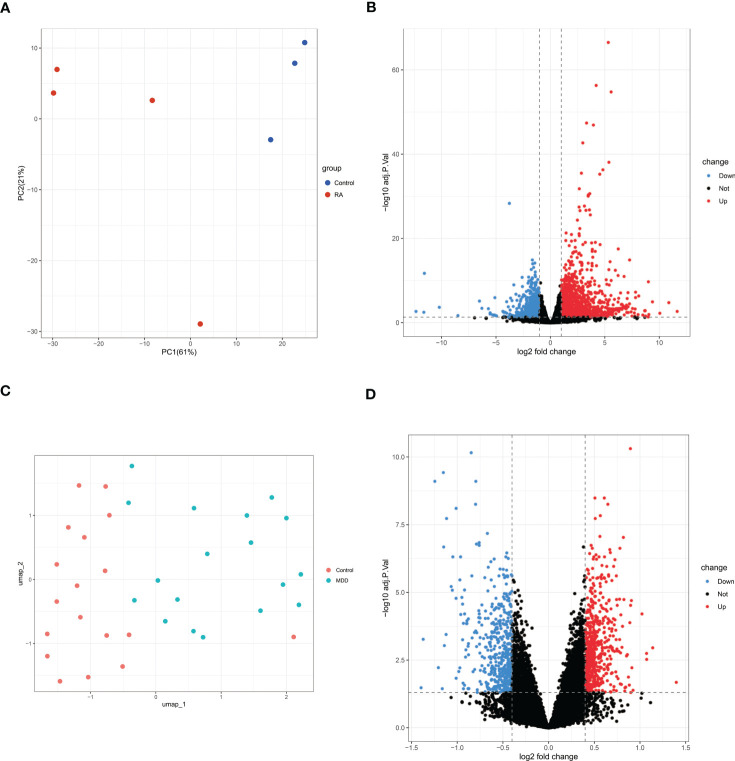
PCA or UMAP and volcano plot for the DEGs identified from RA dataset (GSE169082) and MDD dataset (GSE38206). **(A)**PCA of RA dataset. **(B)** Volcano plot of RA dataset. Red and blue plot dots represent DEGs with upregulated and downregulated gene expression, respectively. **(C)** UMAP of MDD dataset. **(D)** Volcano plot of MDD dataset. PCA, principal component analysis; UMAP, uniform manifold approximation and projection; RA, rheumatoid arthritis; MDD, major depressive disorder; DEGs, differentially expressed genes; GSE, gene expression omnibus series.

A total of 2177 DEGs were identified in GSE169082 using the DESeq2 method, of which 1458 are upregulated and 719 are downregulated. The PCA and volcano plot of RA DEGs are shown in **Figures 3A, B**. The UMAP and volcano plot of MDD DEGs are shown in **Figures 3C, D** (592 upregulated and 441 downregulated).

### 3.3 Protein-protein interaction network construction using enrichment analysis

**Figure 4:**
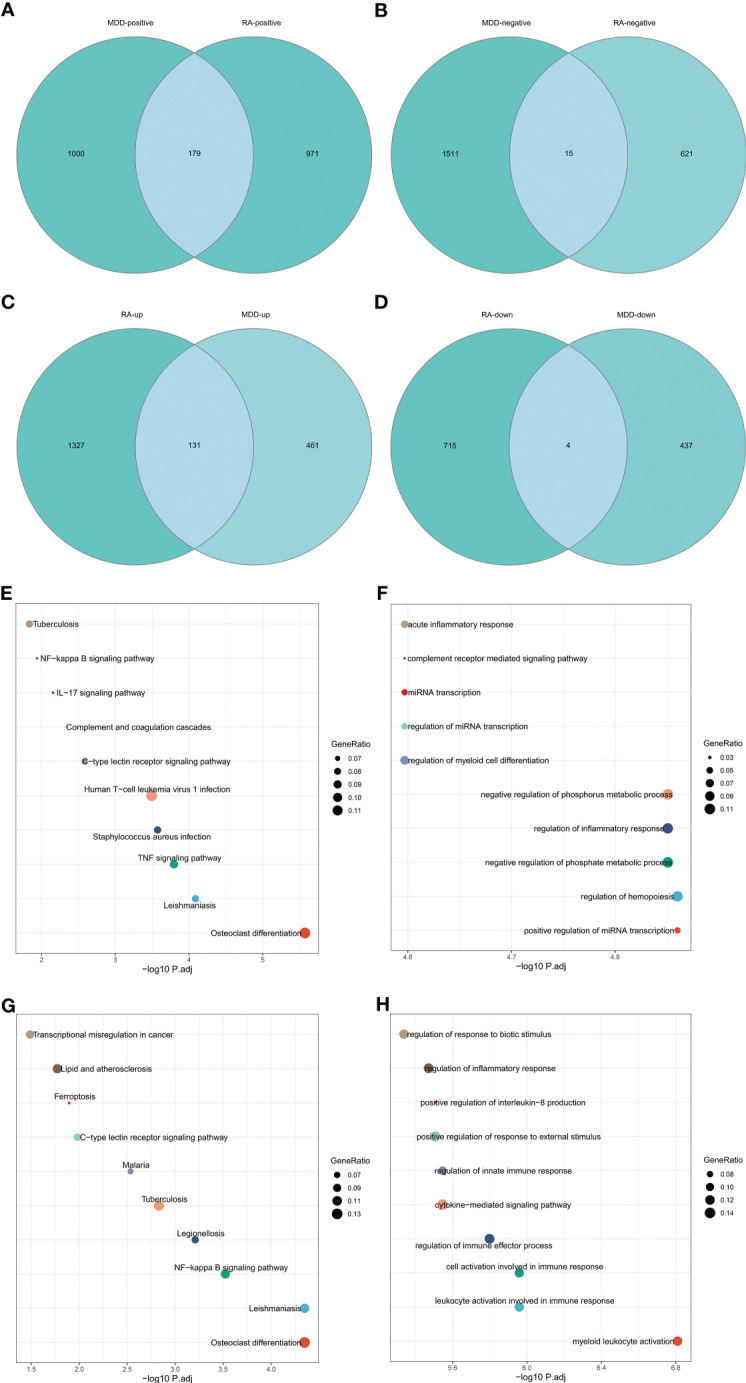
Enrichment analysis. **(A)** Venn diagram shows that 179 genes are identified from the intersection of positive module genes of GSE97476 and GSE38206. **(B)** Venn diagram shows that 15 genes are identified from the intersection of negative module genes of GSE97476 and GSE38206. **(C)** Venn diagram shows that 131 genes are identified from the intersection of up-regulated DEGs of GSE169082 and GSE38206. **(D)** Venn diagram shows that 4 genes are identified from the intersection of down-regulated DEGs of GSE169082 and GSE38206. **(E)** KEGG pathway analysis of the union of 179 and 15 genes. **(F)** GO pathway analysis of the union of 179 and 15 genes. **(G)** KEGG pathway analysis of the union of 131 and 4 genes. **(H)** GO pathway analysis of the union of 131 and 4 genes. KEGG, Kyoto Encyclopedia of Genes and Genomes; GO, Gene Ontology; GSE, gene expression omnibus series; DEGs, differentially expressed genes.

**Figure 5:**
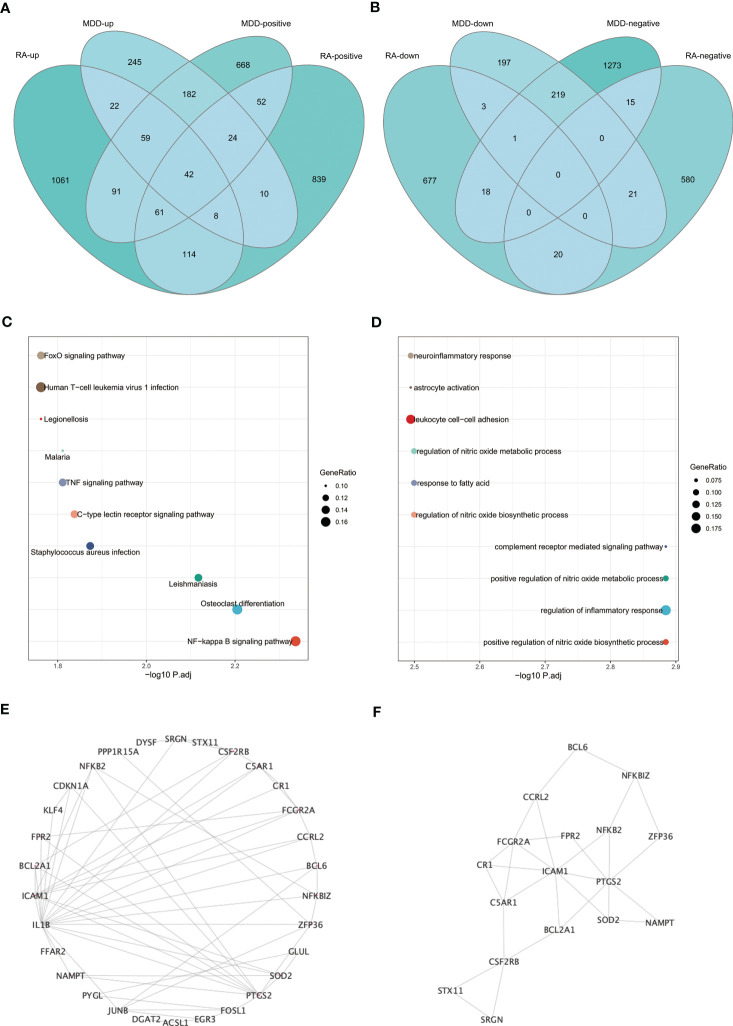
Enrichment analysis and the identification of node genes from PPI network. **(A)** Venn diagram shows that 42 genes are identified from the intersection of positive module genes of GSE97476 and GSE38206 and up-regulated DEGs of GSE169082 and GSE38206. **(B)** Venn diagram shows that 0 genes are identified from the intersection of negative module genes of GSE97476 and GSE38206 and down-regulated DEGs of GSE169082 and GSE38206. **(C)** KEGG pathway analysis of the union of 42 genes. **(D)** GO pathway analysis of the union of 42 genes. **(E)** PPI network reveals that 30 genes interact with each other. **(F)** The subnetworks (17 genes) are screened by MCODE. PPI network, protein-protein interaction network; KEGG, Kyoto Encyclopedia of Genes and Genomes; GO, Gene Ontology; GSE, gene expression omnibus series; DEGs, differentially expressed genes; MCODE, molecular complex detection.

The intersection of positive module genes and the intersection of negative module genes of GSE97476 and GSE38206 are 179 and 15 genes respectively (**Figures 4A, B**), with a total of 194 genes. The intersection of up-regulated DEGs and the intersection of down-regulated DEGs of GSE169082 and GSE38206 were 131 and 4 genes respectively (**Figures 4C, D**), with a total of 194 genes. In addition, we crossed the intersection of the positive module genes of GSE97476 and GSE38206 and the up-regulated DEGs of GSE169082 and GSE38206, and obtained 42 genes (**Figure 5A**); We took the intersection of the negative module gene of GSE97476 and GSE38206 and the intersection of down-regulated DEGs of GSE169082 and GSE38206, and got 0 genes (**Figure 5B**). We performed KEGG analysis and GO analysis on the gene sets of 194,135 and 42 genes respectively (**Figures 4E-H**, **5C, D**). The detailed top ten enrichment ontologies for KEGG and enrichment ontologies for GO are listed in **Tables S1**-**3**. This enrichment analysis revealed that these three gene sets were largely related to immune response and inflammation, which are highly correlated with the pathogenesis of RA and MDD.

To determine whether the screened genes are closely related to immunity, we built an immunity network based on PPIs using String database (https://string-db.org/) to find node proteins that can interact with each other. The node proteins are visualized by Cytoscape software (**Figure 5E**), and the subnetwork is screened by MCODE (**Figure 5F**). The enrichment analysis of 17 proteins in the subnetwork showed that the enrichment pathway was mostly related to immunity and inflammation (**Figure S2**).

### 3.4 Identification of candidate hub genes via machine learning

**Figure 6:**
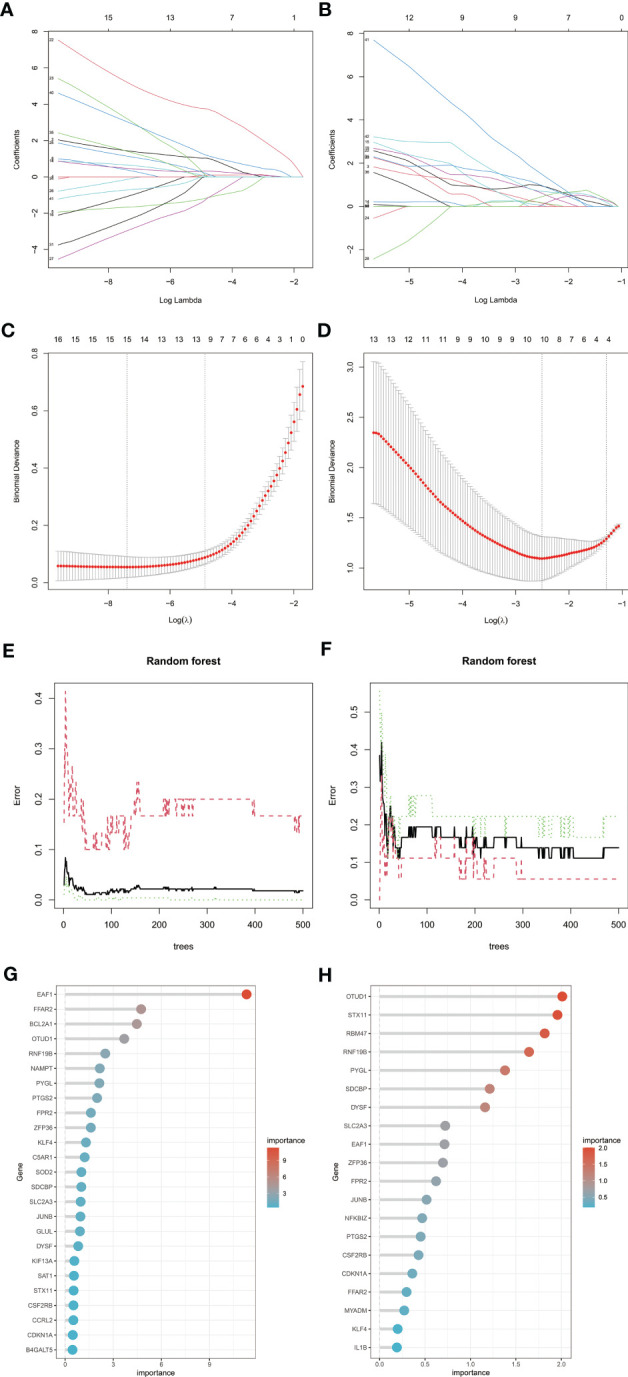
Machine learning in screening candidate diagnostic biomarkers for RA with MDD. **(A, C)** Biomarkers of RA screening in the Lasso model. The number of genes (n = 15) corresponding to the lowest point of the curve is the most suitable for RA diagnosis. **(B, D)** Biomarkers of MDD screening in the Lasso model. The number of genes (n = 10) corresponding to the lowest point of the curve is the most suitable for MDD diagnosis. **(E, G)** The random forest algorithm shows the error in RA; control group and genes are ranked based on the importance score. **(F, H)** The random forest algorithm shows the error in MDD; control group and genes are ranked based on the importance score. RA, rheumatoid arthritis; MDD, major depressive disorder.

To screen candidate genes for nomogram construction and diagnostic value assessment, LASSO regression and RF machine learning algorithms were applied (**Figures 6A–H**). We take the intersection of 15 genes determined by LASSO regression algorithm and the first 25 genes obtained by random forest algorithm as candidate biomarkers of RA, a total of 9 genes. We selected the intersection of 10 genes determined by LASSO regression algorithm and the first 20 genes obtained by random forest algorithm as candidate biomarkers of MDD, a total of 7 genes. Genes were ranked by the RF algorithm based on their importance calculations. The 9 candidate genes of RA and 7 candidate genes of MDD converge into 3 genes (EAF1,SDCBP and RNF19B), which are used for final verification.

### 3.5 Genes assessment

The Wilcoxon test of the three candidate hub genes showed that the three genes were significantly up-regulated in RA and MDD patients. The three candidate hub genes were evaluated by ROC respectively. In RA, the AUC of EAF1, SDCBP, RNF19B is 0.978(95%CI 0.961-0.995), 0.6122(95%CI 0.523-0.702) and 0.942(95%CI 0.909-0.975), respectively. In MDD, the AUC of EAF1, SDCBP, RNF19B is 0.911(95%CI 0.819-1.000), 0.895(95%CI 0.789-1.000) and 0.907(95%CI 0.804-1.000), respectively. The results showed that the higher expression of the three genes, the higher risk of RA or MDD.

**Figure 7:**
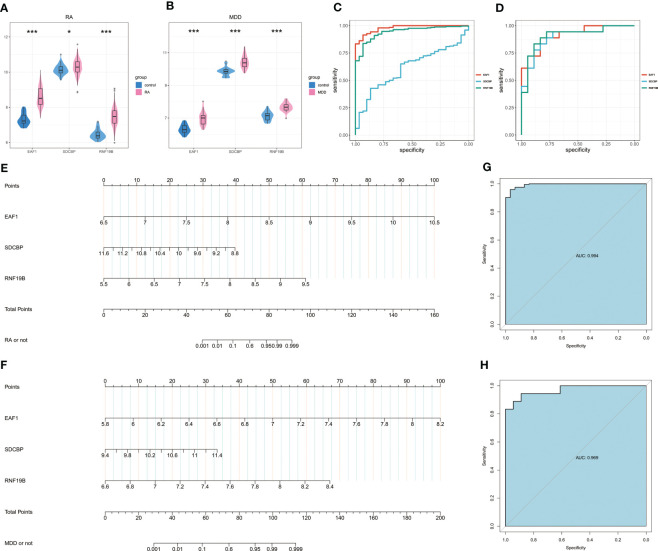
Nomogram construction and the diagnostic value evaluation. **(A)** Expression of EAF1, SDCBP and RNF19B in RA and associated control groups. **(B)** Expression of EAF1, SDCBP and RNF19B in MDD and associated control groups. **(C)** The ROC curve of EAF1, SDCBP and RNF19B in RA. **(D)** The ROC curve of EAF1, SDCBP and RNF19B in MDD. **(E)**The visible nomogram for RA. **(F)** The visible nomogram for MDD. **(G)** The ROC curve shows the significant RA diagnostic value. **(H)** The ROC curve shows the significant MDD diagnostic value. RA, rheumatoid arthritis; MDD, major depressive disorder; ROC curve, receiver operating characteristic curve. * means P-value was below 0.05. *** means P-value was below 0.001.

The nomogram was constructed based on the three candidate hub genes, and a ROC curve was established to assess the diagnostic specificity and sensitivity of the gene set of these three genes and the nomogram (**Figures 7A–D**). We calculated the AUC and 95% CI for each item. The results were as follows: RA(AUC 0.994, 95%CI 0.986-1.000), MDD(AUC 0.969, 95%CI 0.925-1.000). The gene set of these three genes possess a high diagnostic value for RA with MDD, and the constructed nomogram has high diagnostic value.

### 3.6 Immune cell infiltration analysis

Since we observed that RA-associated genes could regulate MDD pathogenesis, were enriched in immune regulation, and could be used as the potential RA and MDD diagnostic biomarker by nomogram construction with ROC evaluation, immune cell infiltration analysis was performed to better elucidate the immune regulation of RA and MDD.

**Figure 8:**
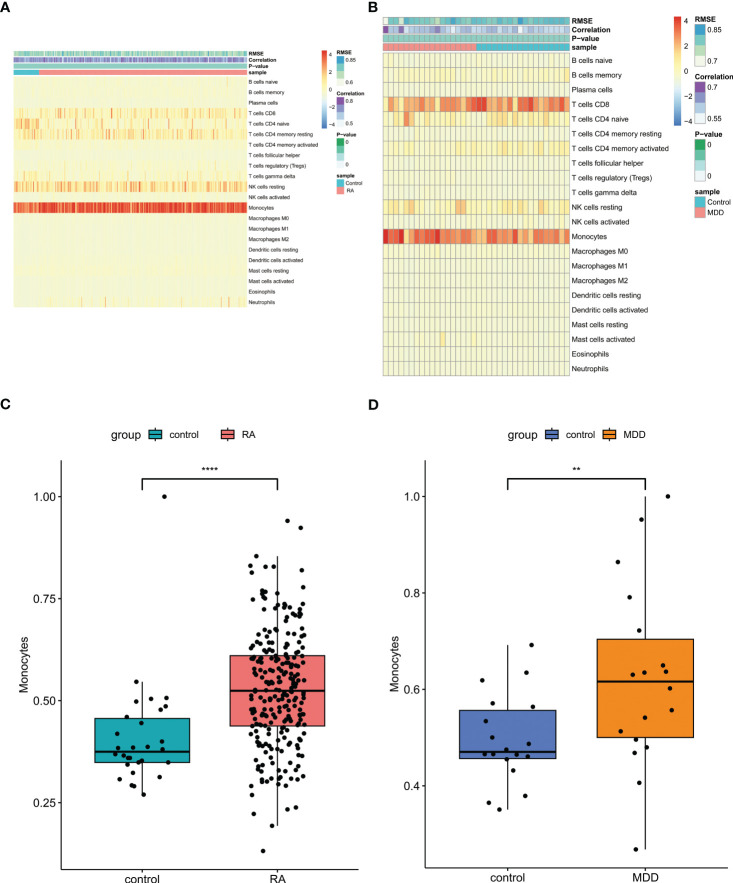
Immune cell infiltration analysis. **(A)** The proportion of 22 kinds of immune cells in different samples of RA and controls visualized from the heatmap. **(B)** The proportion of 22 kinds of immune cells in different samples of MDD and controls visualized from the heatmap. **(C)** Levels of monocytes of RA and controls. **(D)** Levels of monocytes of MDD and controls. RA, rheumatoid arthritis; MDD, major depressive disorder. ** means P-value was below 0.01. **** means P-value was below 0.0001.

For RA and control group or MDD and control group, the scores of 22 kinds of immune cells in each sample were displayed in the heatmap using Cibersort algorithm (**Figures 8A, B**). The patients with RA and MDD had higher levels of monocytes than those in the control group (**Figures 8C, D**).

**Figure 9:**
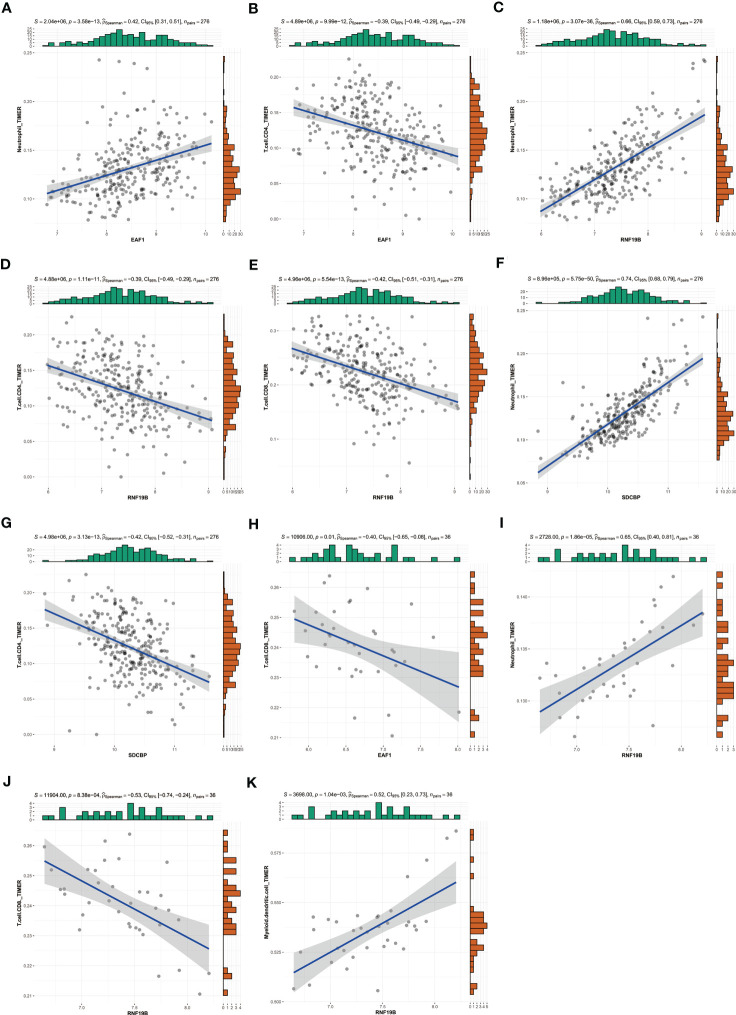
Using TIMER database to explore the potential correlation between EAF1, SDCBP and RNF19B expression and immune cell infiltration. **(A–G)** In patients with RA, EAF1,SDCBP and RNF19B expression had a strong relationship with Neutrophil and CD4+ T cell, and the expression of RNF19B is also closely related to CD8+T cells. **(H–K)** In patients with MDD, EAF1 and RNF19B expression had a strong relationship with CD8+ T cells, and the expression of RNF19B is also closely related to Neutrophil and myeloid dendritic cells. TIMER, Tumor Immune Estimation Resource; RA, rheumatoid arthritis; MDD, major depressive disorder.

In addition, we used TIMER database to explore the potential correlation between EAF1, SDCBP and RNF19B expression and immune cell infiltration. In patients with RA, EAF1, expression of SDCBP and RNF19B have a strong relationship with neutrophils and CD4+ T cells (**Figures 9A–D, F, G**), and the expression of RNF19B is also closely related to CD8+T cells (**Figure 9E**). In patients with MDD, expression of EAF1 and RNF19B have a strong relationship with CD8+ T cells (**Figures 9H, J**), and the expression of RNF19B is also closely related to neutrophils and myeloid dendritic cells (**Figures 9I, K**).

## 4 Discussion

Symptom-based approaches is currently used to diagnose depression. Diagnostic and Statistical Manual of Mental Disorders (DSM) (I to V editions) and International Classification of Diseases (ICD) (from 6th to 11th editions) provide a set of criteria in order to diagnose depression. In spite of this, they are based on the patient’s own description and the clinician’s observation of the patient’s behavior (29). The DSM and ICD do not mention any objective, measurable biological markers that might assist in diagnosing depression. As a result, depression diagnosis can be subjective to a certain degree, which increases the risk of misdiagnosis and suboptimal treatment. Consequently, objective biomarkers for depression are extremely valuable.

There are many explanations for the high frequency of depression in RA patients, including financial stress caused by long-term treatment of chronic diseases, low self-esteem caused by disability, loss of social role caused by unemployment, persistent pain, fatigue, and low energy can also affect the mood of patients. In addition to these psychological causes, recent studies have proposed an inflammation hypothesis to explain possible biological mechanisms (6, 30–32). There is substantial evidence confirming that inflammatory factors are involved in the occurrence and development of depression (8, 33). Elevated levels of inflammatory factors significantly promoted the occurrence of the first depressive episode, and inflammation persisted during the depressive episode (34, 35). The related changes may be used as a diagnostic indicator of depression.

To construct the nomogram and to evaluate the diagnostic value for RA with MDD, we used a bioinformatics analysis and two machine learning methods. The main findings were the identification of three pivotal immune-associated candidate genes (EAF1, SDCBP and RNF19B) and the development of a nomogram for diagnosing RA with MDD. In the study, peripheral blood samples were used for the MDD and RA datasets; for this reason, we only need to collect peripheral blood from patients with RA and evaluate the expression of the three immune-associated genes to infer the likelihood of MDD in RA patients.

ELL-associated Factor 1 (EAF1) was first identified based on its ability to interact with transcriptional elongation factor for RNA polymerase II (36). In lower organisms, the EAF orthologue has been shown to play an important role in growth, DNA damage response and fertility (37, 38), some of which may be achieved by regulating classical or non-classical Wnt/β-catenin signaling pathways (39–41). Disruption of Wnt/β-catenin pathway was observed in several diseases associated with chronic neuroinflammation, such as neurodegenerative diseases (42) and mental disorders such as obsessive-compulsive disorder (43), depression (44) and autism (45). Therefore, EAF1 may control the occurrence and development of depression by regulating Wnt/β-catenin pathway, but there is no direct evidence that there is a causal relationship between EAF1 and depression.

Mda-9/syntenin, encoded by the SDCBP gene, is involved in a variety of functions, including transmembrane protein transport, neural and immune regulation, exocrine biogenesis and human tumorigenesis (46, 47). In VeroE6 cells infected with recombinant severe acute respiratory syndrome coronavirus (SARS-CoV) containing envelope protein, SDCBP interacts with envelope protein to activate p38 MAPK and lead to overexpression of inflammatory cytokines (48). Moreover, SDCBP promotes macrophage migration and angiogenesis in hepatocellular carcinoma by activating NF-κB pathway (49). Signaling pathways, such as p38 MAPK and NF-κB pathways, were all closely related to the occurrence and development of depression (50–52). Hence, the change of SDCBP in PBMC during the occurrence of MDD in patients with RA may control the occurrence of depression through a complex molecular regulatory network and serve as a potential diagnostic index of RA with MDD.

RNF19B, also known as natural killer cell lysis-related molecule(NKLAM), is expressed by a variety of hematopoietic cells, including NK cells, CD8^+^T cells and monocytes (53, 54). In one study, compared with WT mice infected with Sendai virus (SeV), NKLAM^-/-^ mice had less STAT1 and NF-κB p65 phosphorylation in the lungs, thereby reducing the expression of multiple proinflammatory cytokines (55). We speculate that the role of RNF19B in regulating the expression of proinflammatory cytokines may be a bridge between RA and MDD.

The inflammation hypothesis of depression is also known as the monocyte/macrophage theory of depression because monocyte/macrophage lineage cells are important producers of inflammatory cytokines (56). Several studies have found that inflammatory genes are overexpressed in monocytes of patients with MDD (57–59). Moreover, a meta-analysis of seven studies involving 191 cases and 141 controls showed that patients with depression have a higher average absolute monocyte count(60), which is consistent with the results of the immune cell infiltration analysis.

Ziegler-Heitbrock et al. divided human monocytes into three subsets: classical monocyte (CD14^++^CD16^−^), intermediate monocyte (CD14^++^CD16^+^) and non-classical monocyte (CD14^+^CD16^++^) (61). The level of intermediate monocytes is highly up-regulated in peripheral blood and synovial fluid of patients with RA (62–64). However, there are differences in reports of classical and non-classical monocyte levels in RA. Some studies have shown that classical monocyte levels in RA patients are higher than those in normal controls (63), while some reports have shown that non-classical monocyte levels in RA patients are higher than those in healthy controls, and there is no significant difference in classical monocyte levels between RA patients and healthy controls (64). Intermediate monocytes have a substantial role during the inflammatory cascade and have higher capacity to produce and release IL-1β, and TNF-α in response to LPS (65). However, there is still a lack of research on different monocyte subsets of MDD. We speculate that the increase in the number of intermediate monocytes in peripheral blood caused by RA may promote the occurrence and development of MDD.

In conclusion, we used bioinformatics analysis and machine learning to screen three hub genes shared by RA and MDD, including EAF1, SDCBP and RNF19B, which may help to explain the potential molecular mechanism by which RA and MDD increase the morbidity of each other.

## Code availability statement

R scripts for data collation is available for download at https://CRAN.R-project.org/package=tidyverse and https://github.com/slowkow/ggrepel.

R scripts for enrichment analysis is available for download at https://github.com/YuLab-SMU/clusterProfiler and https://bioconductor.org/packages/org.Hs.eg.db/. R scripts for GEO datasets download is available for download at https://bioconductor.org/packages/GEOquery/.

R scripts for PCA analysis is available for download at https://CRAN.R-project.org/package=factoextra and https://CRAN.R-project.org/package=FactoMineR.

R scripts for dimension reduction with UMAP is available for download at https://github.com/lmcinnes/umap.

R scripts for Differential gene analysis is available for download at https://bioconductor.org/packages/limma/ and https://bioconductor.org/packages/DESeq2/.

R scripts for WGCNA is available for download at https://bioconductor.org/packages/GWENA/.

R scripts for drawing of ROC Curves is available for download at https://bioconductor.org/packages/ROC/.

R scripts for drawing of heatmaps is available for download at https://bioconductor.org/packages/heatmaps/.

R scripts for establishment of LASSO regression model is available for download at https://CRAN.R-project.org/package=glmnet.

R scripts for randomForest analysis is available for download at https://CRAN.R-project.org/package=randomForestExplainer.

R scripts for drawing of Venn diagrams is available for download at https://CRAN.R-project.org/package=ggVennDiagram.

## Data availability statement

Publicly available datasets were analyzed in this study. This data can be found here: https://www.ncbi.nlm.nih.gov/geo/query/acc.cgi?acc=GSE97476, https://www.ncbi.nlm.nih.gov/geo/query/acc.cgi?acc=GSE38206, https://www.ncbi.nlm.nih.gov/geo/query/acc.cgi?acc=GSE169082.

## Author contributions

WJ: Conceptualization, Methodology, Data curation, Software, Formal analysis, Visualization, Writing – original draft. XW: Validation, Investigation, Data curation, Formal analysis, Methodology, Writing – original draft. DT: Project administration, Writing – review and editing. XZ: Project administration, Funding acquisition, Resources, Supervision, Writing – review and editing. All authors contributed to the article and approved the submitted version.

## Supplementary material

Supplementary Figure 1 Soft threshold determination of WGCNA networks. **(A)** Soft threshold determination of WGCNA networks of RA dataset. **(B)** Soft threshold determination of WGCNA networks of MDD dataset. WGCNA, weighted gene co-expression network analysis; RA, rheumatoid arthritis; MDD, major depressive disorder.

Supplementary Figure 2 Enrichment analysis of the subnetwork of PPI network. **(A)** KEGG pathway analysis of the subnetwork of PPI network. **(B)** GO pathway analysis of the subnetwork of PPI network.
