# Supplementary figures and images for: Identification of common genetic characteristics of rheumatoid arthritis and major depressive disorder by bioinformatics analysis and machine learning
Source: Front Immunol. 2023 Jun 21;14:1183115. doi: 10.3389/fimmu.2023.1183115 (PMC10320004; doi:10.3389/fimmu.2023.1183115)

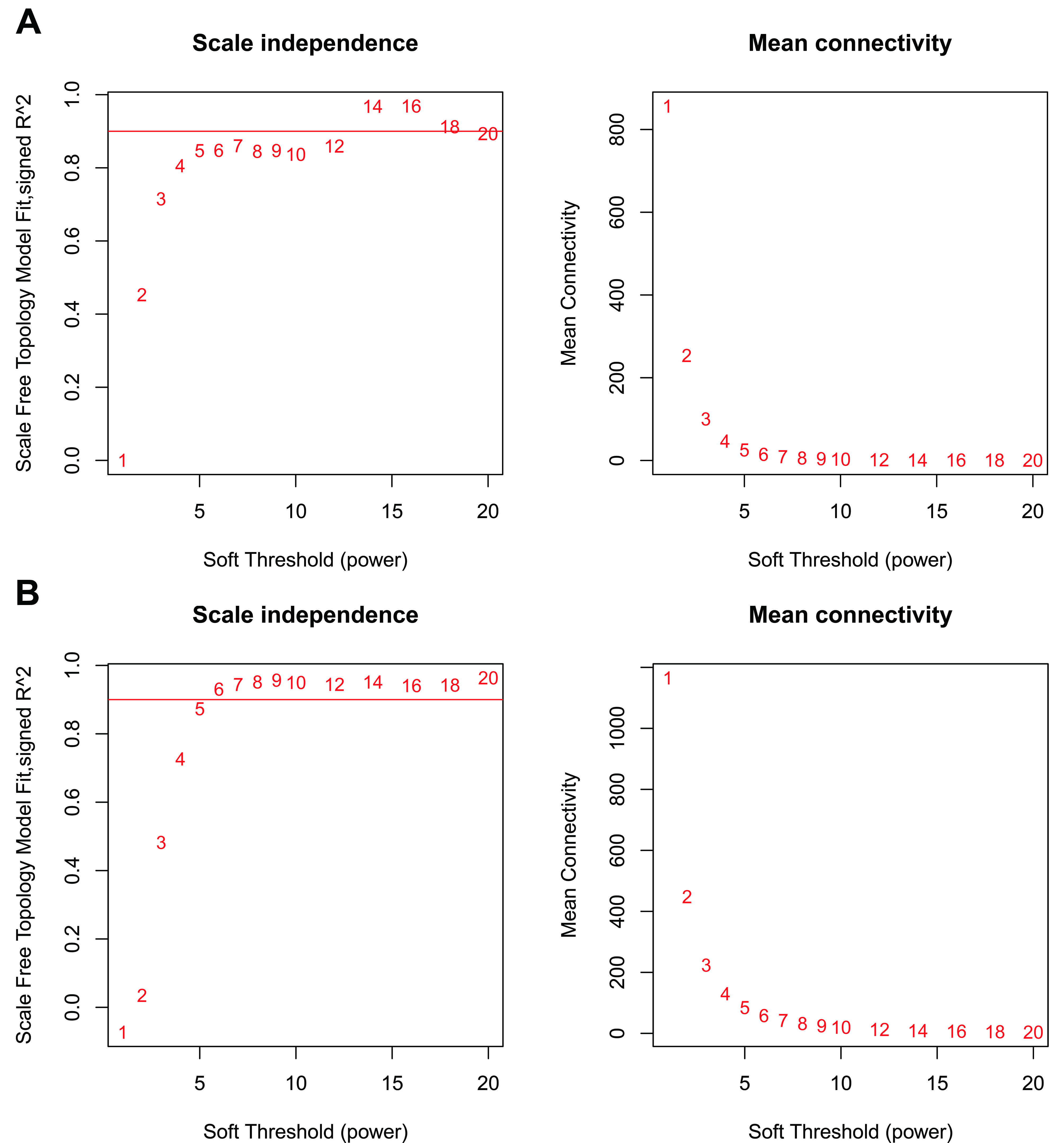

Supplement: Supplementary Figure 1 — Soft threshold determination of WGCNA networks. (A) Soft threshold determination of WGCNA networks of RA dataset. (B) Soft threshold determination of WGCNA networks of MDD dataset. WGCNA, weighted gene co-expression network analysis; RA, rheumatoid arthritis; MDD, major depressive disorder. [file Image_1.tif]

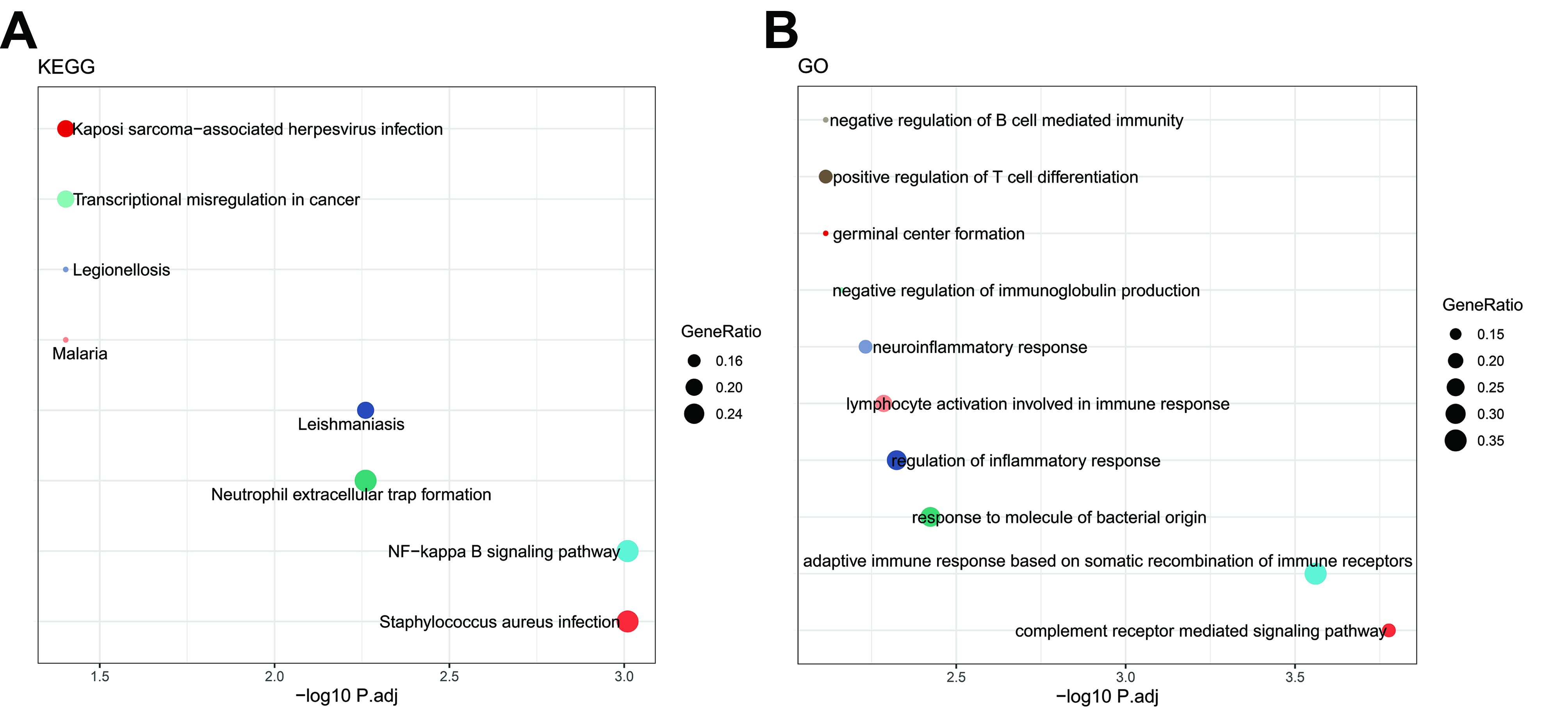

Supplement: Supplementary Figure 2 — Enrichment analysis of the subnetwork of PPI network. (A) KEGG pathway analysis of the subnetwork of PPI network. (B) GO pathway analysis of the subnetwork of PPI network. [file Image_2.tif]
