# Supplementary material for: Identification of common genetic characteristics of rheumatoid arthritis and major depressive disorder by bioinformatics analysis and machine learning
Source: Front Immunol. 2023 Jun 21;14:1183115. doi: 10.3389/fimmu.2023.1183115 (PMC10320004; doi:10.3389/fimmu.2023.1183115)
Supplement: Supplementary file 3 [file Table_1.docx]

| **Supplementary Table S1. Functional enrichment analysis of 194 genes.** | | | | |
| --- | --- | --- | --- | --- |
| ID | Description | Count | p.adjust | Gene ID |
| **KEGG pathway** | | | | |
| hsa04380 | Osteoclast differentiation | 13 | 2.68E-06 | 54/3553/2354/7124/2353/9021/3726/2214/2212/9103/8061/4689/4791 |
| hsa05140 | Leishmaniasis | 9 | 8.19E-05 | 3553/7124/2353/2214/5743/2212/9103/1378/4689 |
| hsa04668 | TNF signaling pathway | 10 | 0.000160328 | 3553/7124/2353/9021/3383/9586/3726/5743/602/1051 |
| hsa05150 | Staphylococcus aureus infection | 9 | 0.000267075 | 2358/719/3383/2214/728/2204/2212/2357/9103 |
| hsa05166 | Human T-cell leukemia virus 1 infection | 13 | 0.000320887 | 7514/1958/1959/7124/2353/3383/9586/7538/1026/8061/4609/4791/2113 |
| hsa04625 | C-type lectin receptor signaling pathway | 8 | 0.002614424 | 3553/1959/7124/5743/602/1960/114548/4791 |
| hsa04610 | Complement and coagulation cascades | 7 | 0.004562587 | 719/5329/728/7056/1378/1604/3687 |
| hsa04657 | IL-17 signaling pathway | 7 | 0.006972606 | 3553/2354/7124/2353/5743/8061/1051 |
| hsa04064 | NF-kappa B signaling pathway | 7 | 0.011540952 | 3553/7124/3383/5743/597/4791/4616 |
| hsa05152 | Tuberculosis | 9 | 0.014588886 | 3553/7124/2214/2212/9103/7096/1378/1051/3687 |
| **Biological Process** | | | | |
| GO:1902895 | positive regulation of miRNA transcription | 8 | 1.38E-05 | HIF1A/EGR1/TNF/FOS/KLF4/FOSL1/MYC/ETS1 |
| GO:1903706 | regulation of hemopoiesis | 18 | 1.38E-05 | HIF1A/TOB2/TNF/FOS/MAFB/KLF10/TRIB1/ZFP36/EGR3/BCL6/NLRP3/CR1/CEBPB/CSF3R/NFKBIZ/TNFAIP6/MYC/ETS1 |
| GO:0045936 | negative regulation of phosphate metabolic process | 19 | 1.41E-05 | UBE2B/IL1B/TNF/DUSP6/SOCS3/DUSP1/TRIB1/LRRK2/C9orf72/PPP1R15A/CDKN1A/IRAK3/RGS2/MYADM/CDA/SAMSN1/MIDN/GNAQ/GADD45B |
| GO:0050727 | regulation of inflammatory response | 18 | 1.41E-05 | ACP5/IL1B/TNF/FPR2/FFAR2/SOCS3/KLF4/LRRK2/ZFP36/LDLR/PTGS2/BCL6/NLRP3/CEBPB/NFKBIZ/TNFAIP6/DNASE1L3/ETS1 |
| GO:0010563 | negative regulation of phosphorus metabolic process | 19 | 1.41E-05 | UBE2B/IL1B/TNF/DUSP6/SOCS3/DUSP1/TRIB1/LRRK2/C9orf72/PPP1R15A/CDKN1A/IRAK3/RGS2/MYADM/CDA/SAMSN1/MIDN/GNAQ/GADD45B |
| GO:0045637 | regulation of myeloid cell differentiation | 13 | 2.53E-05 | HIF1A/TOB2/TNF/FOS/MAFB/KLF10/TRIB1/ZFP36/CEBPB/CSF3R/TNFAIP6/MYC/ETS1 |
| GO:1902893 | regulation of miRNA transcription | 8 | 2.53E-05 | HIF1A/EGR1/TNF/FOS/KLF4/FOSL1/MYC/ETS1 |
| GO:0061614 | miRNA transcription | 8 | 2.53E-05 | HIF1A/EGR1/TNF/FOS/KLF4/FOSL1/MYC/ETS1 |
| GO:0002430 | complement receptor mediated signaling pathway | 5 | 2.53E-05 | FPR2/C3AR1/C5AR1/FPR1/CR1 |
| GO:0002526 | acute inflammatory response | 10 | 2.53E-05 | ORM1/IL1B/TNF/FFAR2/TREM1/FCGR3A/PTGS2/NLRP3/CEBPB/DNASE1L3 |
| **Cellular Component** | | | | |
| GO:0070820 | tertiary granule | 13 | 6.65E-07 | ORM1/FPR2/SIGLEC5/LRG1/FCAR/FPR1/CR1/CDA/SLC2A3/TMEM63A/TNFAIP6/CD55/ITGAX |
| GO:0101002 | ficolin-1-rich granule | 13 | 1.44E-06 | FPR2/SIGLEC5/LRG1/MNDA/FCAR/FPR1/PYGL/CR1/CDA/SLC2A3/TNFAIP6/CD55/ITGAX |
| GO:0030667 | secretory granule membrane | 16 | 1.69E-06 | FPR2/ANPEP/C3AR1/SIGLEC5/PLAUR/C5AR1/FCAR/FCGR2A/FPR1/CR1/SLC2A3/STX3/TMEM63A/CD55/P2RX1/ITGAX |
| GO:0101003 | ficolin-1-rich granule membrane | 8 | 4.25E-06 | FPR2/SIGLEC5/FCAR/FPR1/CR1/SLC2A3/CD55/ITGAX |
| GO:0042581 | specific granule | 10 | 8.85E-05 | ORM1/FPR2/C3AR1/PLAUR/LRG1/FCAR/SLC2A3/STX3/TMEM63A/P2RX1 |
| GO:0035579 | specific granule membrane | 7 | 0.000733917 | FPR2/C3AR1/PLAUR/FCAR/SLC2A3/TMEM63A/P2RX1 |
| GO:0070821 | tertiary granule membrane | 6 | 0.001773732 | FPR2/SIGLEC5/FCAR/SLC2A3/TMEM63A/ITGAX |
| GO:0005766 | primary lysosome | 8 | 0.002185965 | C3AR1/MNDA/FPR1/GM2A/GCA/SDCBP/CPPED1/STX3 |
| GO:0042582 | azurophil granule | 8 | 0.002185965 | C3AR1/MNDA/FPR1/GM2A/GCA/SDCBP/CPPED1/STX3 |
| GO:0034774 | secretory granule lumen | 11 | 0.003686685 | ORM1/S100P/LRG1/MNDA/GM2A/GCA/PYGL/SDCBP/CDA/CPPED1/SRGN |
| **Molecular Function** | | | | |
| GO:0004875 | complement receptor activity | 5 | 2.11E-05 | FPR2/C3AR1/C5AR1/FPR1/CR1 |
| GO:0140375 | immune receptor activity | 10 | 0.000273625 | FPR2/C3AR1/KLRC1/FCGR3A/C5AR1/FPR1/CR1/CSF3R/CSF2RB/CCRL2 |
| GO:0001228 | DNA-binding transcription activator activity, RNA polymerase II-specific | 16 | 0.00084054 | HIF1A/EGR1/FOSB/EGR2/FOS/MAFB/KLF4/KLF10/JUNB/EGR3/FOSL1/CEBPB/ATF3/MYC/NFKB2/ETS1 |
| GO:0001216 | DNA-binding transcription activator activity | 16 | 0.00084054 | HIF1A/EGR1/FOSB/EGR2/FOS/MAFB/KLF4/KLF10/JUNB/EGR3/FOSL1/CEBPB/ATF3/MYC/NFKB2/ETS1 |
| GO:0019865 | immunoglobulin binding | 4 | 0.005647633 | FCGR3A/FCAR/FCGR2A/FCGR2C |
| GO:0019864 | IgG binding | 3 | 0.008718068 | FCGR3A/FCGR2A/FCGR2C |
| GO:0140297 | DNA-binding transcription factor binding | 13 | 0.029031649 | HIF1A/TOB2/EGR2/FOS/KLF4/TRIB1/DTX3L/BCL3/BCL6/NLRP3/CEBPB/MYC/ETS1 |
| GO:0001046 | core promoter sequence-specific DNA binding | 4 | 0.032228529 | FOS/KLF10/CEBPB/MYC |
| GO:0001618 | virus receptor activity | 5 | 0.032228529 | ANPEP/ICAM1/LDLR/CR1/CD55 |
| GO:0140272 | exogenous protein binding | 5 | 0.032228529 | ANPEP/ICAM1/LDLR/CR1/CD55 |
