# Supplementary material for: Identification of common genetic characteristics of rheumatoid arthritis and major depressive disorder by bioinformatics analysis and machine learning
Source: Front Immunol. 2023 Jun 21;14:1183115. doi: 10.3389/fimmu.2023.1183115 (PMC10320004; doi:10.3389/fimmu.2023.1183115)
Supplement: Supplementary file 4 [file Table_2.docx]

| **Supplementary Table S2. Functional enrichment analysis of 135 genes.** | | | | |
| --- | --- | --- | --- | --- |
| ID | Description | Count | p.adjust | Gene ID |
| **KEGG pathway** | | | | |
| hsa04380 | Osteoclast differentiation | 10 | 3.93E-05 | 140885/4791/3460/11027/3553/653361/2212/3726/6688/8061 |
| hsa05140 | Leishmaniasis | 8 | 3.93E-05 | 3460/3553/653361/4615/5743/2212/1378/7097 |
| hsa04064 | NF-kappa B signaling pathway | 8 | 0.000262618 | 4791/3383/3553/4615/5743/4067/597/4055 |
| hsa05134 | Legionellosis | 6 | 0.000542136 | 4791/3553/4615/1378/7097/3310 |
| hsa05152 | Tuberculosis | 9 | 0.001298806 | 3460/3553/4615/2212/1378/7097/972/2207/1509 |
| hsa05144 | Malaria | 5 | 0.002563183 | 3383/3553/4615/1378/7097 |
| hsa04625 | C-type lectin receptor signaling pathway | 6 | 0.009190495 | 4791/3553/3659/1960/5743/2207 |
| hsa04216 | Ferroptosis | 4 | 0.011186704 | 6303/2512/2495/2180 |
| hsa05417 | Lipid and atherosclerosis | 8 | 0.014843861 | 3383/3553/653361/4615/6648/4067/7097/3310 |
| hsa05202 | Transcriptional misregulation in cancer | 7 | 0.028616908 | 1026/1050/6688/604/64332/597/6692 |
| **Biological Process** | | | | |
| GO:0002274 | myeloid leukocyte activation | 15 | 1.55E-07 | IFNGR2/DYSF/LILRA2/STXBP2/CEBPA/MYD88/SPI1/LGALS9/FPR2/LYN/C5AR1/TLR2/CD74/FCER1G/LTBR |
| GO:0002366 | leukocyte activation involved in immune response | 15 | 1.10E-06 | ICAM1/DYSF/LILRA2/STXBP2/MYD88/SPI1/LGALS9/BCL6/CR1/NFKBID/NFKBIZ/LYN/CD74/FCER1G/ZNF683 |
| GO:0002263 | cell activation involved in immune response | 15 | 1.10E-06 | ICAM1/DYSF/LILRA2/STXBP2/MYD88/SPI1/LGALS9/BCL6/CR1/NFKBID/NFKBIZ/LYN/CD74/FCER1G/ZNF683 |
| GO:0002697 | regulation of immune effector process | 16 | 1.59E-06 | ICAM1/IL1B/STXBP2/NCF1/FCN1/SPI1/AHR/LGALS9/BCL6/CR1/NFKBID/NFKBIZ/LYN/FFAR2/CD74/ZNF683 |
| GO:0019221 | cytokine-mediated signaling pathway | 18 | 2.84E-06 | PARP9/IFNGR2/LILRA2/IL1B/IRF1/CEBPA/MYD88/CSF2RB/CSF2RA/SPI1/HCK/NR1H2/CCR1/CD74/FCER1G/ACSL1/CCRL2/PADI2 |
| GO:0045088 | regulation of innate immune response | 13 | 2.84E-06 | PARP9/LILRA2/IRF1/NCF1/FCN1/SPI1/LGALS9/CR1/FPR2/HCK/LYN/NR1H2/FFAR2 |
| GO:0032103 | positive regulation of response to external stimulus | 17 | 3.11E-06 | PARP9/LILRA2/IL1B/CEBPA/PTGS2/FCN1/SPI1/LGALS9/FPR2/NFKBIZ/HCK/LYN/FFAR2/C5AR1/TLR2/CCR1/CD74 |
| GO:0032757 | positive regulation of interleukin-8 production | 8 | 3.11E-06 | LILRA2/IL1B/MYD88/FCN1/LGALS9/FFAR2/TLR2/CD74 |
| GO:0050727 | regulation of inflammatory response | 16 | 3.38E-06 | SIRPA/IL1B/NCF1/CEBPA/MYD88/PTGS2/BCL6/FPR2/NFKBIZ/HCK/LYN/KLF4/FFAR2/ZFP36/TLR2/ZYX |
| GO:0002831 | regulation of response to biotic stimulus | 15 | 4.59E-06 | PARP9/LILRA2/IL1B/IRF1/NCF1/FCN1/SPI1/AHR/LGALS9/CR1/FPR2/HCK/LYN/NR1H2/FFAR2 |
| **Cellular Component** | | | | |
| GO:0101002 | ficolin-1-rich granule | 12 | 1.11E-06 | CRISPLD2/QPCT/SIRPA/FCN1/SLC2A3/CR1/FPR2/FTH1/FCER1G/HSPA6/PYGL/CTSD |
| GO:0070820 | tertiary granule | 10 | 1.62E-05 | QPCT/SIRPA/STXBP2/SLC2A3/CR1/FPR2/FTH1/FCER1G/PTX3/CTSD |
| GO:0034774 | secretory granule lumen | 13 | 1.62E-05 | CRISPLD2/QPCT/FCN1/SRGN/FTL/GCA/SDCBP/HSPA6/S100A11/PTX3/PYGL/CTSD/PADI2 |
| GO:0060205 | cytoplasmic vesicle lumen | 13 | 1.62E-05 | CRISPLD2/QPCT/FCN1/SRGN/FTL/GCA/SDCBP/HSPA6/S100A11/PTX3/PYGL/CTSD/PADI2 |
| GO:0031983 | vesicle lumen | 13 | 1.62E-05 | CRISPLD2/QPCT/FCN1/SRGN/FTL/GCA/SDCBP/HSPA6/S100A11/PTX3/PYGL/CTSD/PADI2 |
| GO:0030667 | secretory granule membrane | 12 | 5.56E-05 | SIRPA/FCGR2A/MGST1/SLC2A3/CR1/FPR2/C5AR1/CKAP4/TLR2/CD68/APLP2/FCER1G |
| GO:0005766 | primary lysosome | 8 | 0.000322148 | STXBP2/MGST1/FTL/GCA/CKAP4/CD68/SDCBP/PADI2 |
| GO:0042582 | azurophil granule | 8 | 0.000322148 | STXBP2/MGST1/FTL/GCA/CKAP4/CD68/SDCBP/PADI2 |
| GO:1904813 | ficolin-1-rich granule lumen | 7 | 0.000588679 | CRISPLD2/QPCT/FCN1/FTH1/HSPA6/PYGL/CTSD |
| GO:0005775 | vacuolar lumen | 8 | 0.000596695 | VCAN/FTL/GCA/TCN2/CD74/SDCBP/CTSD/PADI2 |
| **Molecular Function** | | | | |
| GO:0140375 | immune receptor activity | 11 | 4.26E-06 | IFNGR2/LILRA2/CSF2RB/CSF2RA/CR1/FPR2/C5AR1/CCR1/CD74/FCER1G/CCRL2 |
| GO:0004896 | cytokine receptor activity | 6 | 0.008099352 | IFNGR2/CSF2RB/CSF2RA/CCR1/CD74/CCRL2 |
| GO:0004875 | complement receptor activity | 3 | 0.008099352 | CR1/FPR2/C5AR1 |
| GO:0097677 | STAT family protein binding | 3 | 0.008099352 | PARP9/CEBPA/SPI1 |
| GO:0019865 | immunoglobulin binding | 3 | 0.046622476 | LILRA2/FCGR2A/FCER1G |
| GO:0019957 | C-C chemokine binding | 3 | 0.046622476 | ZFP36/CCR1/CCRL2 |
