# Supplementary material for: Identification of common genetic characteristics of rheumatoid arthritis and major depressive disorder by bioinformatics analysis and machine learning
Source: Front Immunol. 2023 Jun 21;14:1183115. doi: 10.3389/fimmu.2023.1183115 (PMC10320004; doi:10.3389/fimmu.2023.1183115)
Supplement: Supplementary file 5 [file Table_3.docx]

| **Supplementary Table S3. Functional enrichment analysis of 42 genes.** | | | | |
| --- | --- | --- | --- | --- |
| ID | Description | Count | p.adjust | Gene ID |
| **KEGG pathway** | | | | |
| hsa04064 | NF-kappa B signaling pathway | 5 | 0.00395537 | 3553/3383/5743/597/4791 |
| hsa04380 | Osteoclast differentiation | 5 | 0.005352061 | 3553/3726/2212/8061/4791 |
| hsa05140 | Leishmaniasis | 4 | 0.006551941 | 3553/5743/2212/1378 |
| hsa05150 | Staphylococcus aureus infection | 4 | 0.011495542 | 2358/3383/728/2212 |
| hsa04625 | C-type lectin receptor signaling pathway | 4 | 0.012473926 | 3553/5743/1960/4791 |
| hsa04668 | TNF signaling pathway | 4 | 0.013259454 | 3553/3383/3726/5743 |
| hsa05144 | Malaria | 3 | 0.013259454 | 3553/3383/1378 |
| hsa05134 | Legionellosis | 3 | 0.014842042 | 3553/1378/4791 |
| hsa05166 | Human T-cell leukemia virus 1 infection | 5 | 0.014842042 | 3383/7538/1026/8061/4791 |
| hsa04068 | FoxO signaling pathway | 4 | 0.014842042 | 6446/6648/1026/604 |
| **Biological Process** | | | | |
| GO:0045429 | positive regulation of nitric oxide biosynthetic process | 4 | 0.001304242 | IL1B/KLF4/SOD2/PTGS2 |
| GO:0050727 | regulation of inflammatory response | 8 | 0.001304242 | IL1B/FPR2/FFAR2/KLF4/ZFP36/PTGS2/BCL6/NFKBIZ |
| GO:1904407 | positive regulation of nitric oxide metabolic process | 4 | 0.001304242 | IL1B/KLF4/SOD2/PTGS2 |
| GO:0002430 | complement receptor mediated signaling pathway | 3 | 0.001304242 | FPR2/C5AR1/CR1 |
| GO:0045428 | regulation of nitric oxide biosynthetic process | 4 | 0.003166453 | IL1B/KLF4/SOD2/PTGS2 |
| GO:0070542 | response to fatty acid | 4 | 0.003166453 | FFAR2/ACSL1/PTGS2/DGAT2 |
| GO:0080164 | regulation of nitric oxide metabolic process | 4 | 0.003166453 | IL1B/KLF4/SOD2/PTGS2 |
| GO:0002460 | adaptive immune response based on somatic recombination of immune receptors built from immunoglobulin superfamily domains | 7 | 0.003204155 | IL1B/ICAM1/BCL6/CR1/CSF2RB/NFKBIZ/NFKB2 |
| GO:0007159 | leukocyte cell-cell adhesion | 7 | 0.003204155 | IL1B/KLF4/ICAM1/EGR3/BCL6/CR1/NFKBIZ |
| GO:0048143 | astrocyte activation | 3 | 0.003204155 | IL1B/FPR2/C5AR1 |
| **Cellular Component** | | | | |
| GO:0101003 | ficolin-1-rich granule membrane | 3 | 0.011957592 | FPR2/CR1/SLC2A3 |
| GO:0044194 | cytolytic granule | 2 | 0.011957592 | SRGN/RNF19B |
| GO:0030667 | secretory granule membrane | 5 | 0.011957592 | FPR2/C5AR1/FCGR2A/CR1/SLC2A3 |
| GO:0045121 | membrane raft | 5 | 0.011957592 | ICAM1/PTGS2/MYADM/CR1/SDCBP |
| GO:0098857 | membrane microdomain | 5 | 0.011957592 | ICAM1/PTGS2/MYADM/CR1/SDCBP |
| GO:0101002 | ficolin-1-rich granule | 4 | 0.011957592 | FPR2/PYGL/CR1/SLC2A3 |
| GO:0031968 | organelle outer membrane | 4 | 0.021470542 | ACSL1/PTGS2/PPP1R15A/BCL2A1 |
| GO:0019867 | outer membrane | 4 | 0.021470542 | ACSL1/PTGS2/PPP1R15A/BCL2A1 |
| GO:0034774 | secretory granule lumen | 4 | 0.049010883 | GCA/PYGL/SDCBP/SRGN |
| GO:0060205 | cytoplasmic vesicle lumen | 4 | 0.049010883 | GCA/PYGL/SDCBP/SRGN |
| **Molecular Function** | | | | |
| GO:0004875 | complement receptor activity | 3 | 0.000414245 | FPR2/C5AR1/CR1 |
| GO:0140375 | immune receptor activity | 5 | 0.001816753 | FPR2/C5AR1/CR1/CSF2RB/CCRL2 |
| GO:0005536 | glucose binding | 2 | 0.01590646 | PYGL/SLC2A3 |
